# PClass: Protein Quaternary Structure Classification by Using Bootstrapping Strategy as Model Selection

**DOI:** 10.3390/genes9020091

**Published:** 2018-02-14

**Authors:** Chi-Chou Huang, Chi-Chang Chang, Chi-Wei Chen, Shao-yu Ho, Hsung-Pin Chang, Yen-Wei Chu

**Affiliations:** 1 School of Medicine, Chung Shan Medical University, Taichung 40201, Taiwan; hcjy341@ms1.hinet.net; 2 Division of Colon & Rectal Surgery, Department of Surgery, Chung Shan Medical University Hospital, Taichung 40201, Taiwan; 3 School of Medical Informatics, Chung-Shan Medical University, Taichung 40201, Taiwan; threec@csmu.edu.tw; 4 IT Office, Chung Shan Medical University Hospital, Taichung 40201, Taiwan; 5 Institute of Genomics and Bioinformatics, National Chung Hsing University, Kuo Kuang Rd., Taichung 402, Taiwan; d103056006@mail.nchu.edu.tw (C.-W.C.); jjkoko916@hotmail.com (S.-y.H.); 6 Department of Computer Science and Engineering, National Chung-Hsing University, Kuo Kuang Rd., Taichung 402, Taiwan; hpchang@cs.nchu.edu.tw; 7 Biotechnology Center, Agricultural Biotechnology Center, Institute of Molecular Biology, Graduate Institute of Biotechnology, National Chung Hsing University, Kuo Kuang Rd., Taichung 402, Taiwan

**Keywords:** protein quaternary structure, bootstrap strategy, model selection, classification

## Abstract

Protein quaternary structure complex is also known as a multimer, which plays an important role in a cell. The dimer structure of transcription factors is involved in gene regulation, but the trimer structure of virus-infection-associated glycoproteins is related to the human immunodeficiency virus. The classification of the protein quaternary structure complex for the post-genome era of proteomics research will be of great help. Classification systems among protein quaternary structures have not been widely developed. Therefore, we designed the architecture of a two-layer machine learning technique in this study, and developed the classification system PClass. The protein quaternary structure of the complex is divided into five categories, namely, monomer, dimer, trimer, tetramer, and other subunit classes. In the framework of the bootstrap method with a support vector machine, we propose a new model selection method. Each type of complex is classified based on sequences, entropy, and accessible surface area, thereby generating a plurality of feature modules. Subsequently, the optimal model of effectiveness is selected as each kind of complex feature module. In this stage, the optimal performance can reach as high as 70% of Matthews correlation coefficient (MCC). The second layer of construction combines the first-layer module to integrate mechanisms and the use of six machine learning methods to improve the prediction performance. This system can be improved over 10% in MCC. Finally, we analyzed the performance of our classification system using transcription factors in dimer structure and virus-infection-associated glycoprotein in trimer structure. PClass is available via a web interface at http://predictor.nchu.edu.tw/PClass/.

## 1. Introduction

The most important intracellular signaling process requires polymerization into a multimer structure by the protein monomer structure to complete cell regulation and active function. However, many proteins can function as a monomer structure, such as enzymes, which can bind with a substrate to enhance the combination of other subunits and accelerate their reaction [1]. Protein complexes are usually described by the number of subunits. A complex with two subunits is called a dimer, which includes transcription factors [2], cell receptors [3], and cytoskeleton proteins [4]. The trimer structure contains three subunits, such as collagen [5], virus-infection-associated glycoproteins [6], and hemagglutinin [7]. A tetramer contains four subunits, such as immunoglobulin protein [8], hemoglobin [9], and avidin [10]. A hexamer contains six subunits, such as the DnaB helicase [11], serum protein [12], and insulin [13,14]. An octamer contains eight subunits, such as earthworm’s serum albumin (hemerythrin) [15] and nucleosome [16]. Under normal circumstances, the protein complexes in cells are rarely more than an octamer, but some exceptions include the proteasome, spliceosome, and exosome. Therefore, monomers and multimers play an important role in biological cells—and also they may lead to cancer and the development of new drugs [17,18,19,20,21].

To comprehend how a polymer is formed, polyacrylamide gel electrophoresis [22], mass spectrometry [23], high performance liquid chromatography (HPLC)-gel filtration chromatography [24], analytical ultracentrifugation [25], and multi-angle laser light scattering [26] analyses are usually conducted to determine the size and distribution of the polymer. However, such experimental methods may be time consuming, laborious, and costly. The development of an in silico method for protein quaternary structure complexes may assist biological experiments.

However, only two studies presented the use of machine learning to determine the protein quaternary structure complex. Multicoil [27] utilizes the covariance matrix of a multivariate Gaussian distribution to predict whether a coiled coil sequence belongs to a dimeric coiled coil, trimeric coiled coil, or noncoiled coil structure. Each residue is given a predicted score. Multicoil2 combines Multicoil with multinomial logistic regression to obtain two predictors of dimer and trimer propensity. These predictors are used to generate potentials for a Markov random field. SCORER [28] uses the log-odds-based scoring system to differentiate between a parallel dimeric coiled coil or parallel trimeric coiled coil. SCORER 2.0 [29] improves the log-odds-based scoring system, makes good use of position-specific scoring matrix and Multicoil, and predicts parallel coiled coil sequence of hepetad repeat location and gives it a score. High scores represent high accuracy in predicting dimer or trimer structures.

Few studies have focused on the dimer and trimer structures, and complete protein quaternary structure complex bioinformatics tools are lacking. This study established a protein quaternary structure complex prediction system by designing a two-layer machine learning framework, and optimal classification and prediction system PClass. The first layer, using the bootstrap method, proposed a new model selection with amino acid sequence composition, entropy, and accessible surface area (ASA) as feature coding. Support vector machine (SVM) was used to select the best performance learning module to build a feature module, wherein the prediction performance was able to be as high as 70% of a Matthews correlation coefficient (MCC). Subsequently, we selected the best feature model to establish a second-layer prediction model, which was integrated by the first layer through machine learning for model selection and prediction of protein quaternary structure complex. The MCC ranged from 70% to 80%. To further investigate the accuracy of the protein quaternary structure complex prediction system, we used dimer-structured transcription factors, and virus-infection-associated glycoproteins in which have a trimer structure as a classification system to verify the protein quaternary structure complex. Finally, the prediction accuracy of the classification system was determined to reach 66% of accuracy (ACC).

## 2. Materials and Methods 

### 2.1. Dataset

As mentioned above, to examine the complete protein quaternary structure complex, this study integrated two different databases, namely, the coiled-coil sequence location database and protein complex structure database, to create and verify the prediction system. One of the datasets was CC + DATABASE [30], which Testa et al. proposed after adjusting the coiled-coil structure and polymer data. In SCORER 2.0 web server, they also utilized the CC + DATABASE but only the parallel dimer and trimer coiled-coil data. In the present study, we used the dataset of all polymers in the CC + DATABASE and classified the polymers into four categories (dimer, trimer, tetramer, and other subunits). Another dataset of the 3D complex was used [31], which was proposed as a protein complex structure database. The 3D complex provides a protein domain structure, cell expression system, accessible surface area of the complex, subunit type, and homologous and heterologous polymers.

In this study, we analysed the database of homologous or heterologous monomers and polymers, classified the data into five categories (monomer, dimer, trimer, tetramer, and other subunits), and integrated the data with the CC + DATABASE for the study dataset. We used data from 2007 and 2006 to verify the established system, whereas data from the years before 2006 were the basis for the establishment of the monomer and polymer system module (Table 1). The study dataset was divided into five categories, and monomers were classified as positive. The remaining non-monomer data were categorized as negative information. The other polymers are shown in Table 2.

### 2.2. Feature Encoding

#### 2.2.1. Amino Acid Composition

Amino acid composition (AAC) describes the basic unit of a protein, which has specific molecular structure patterns, such as charge, size, polarity, and solubility (hydrophilic and hydrophobic). Proteins have biochemical activity. Therefore, we used 20 kinds of amino acids in the composition and the other remaining amino acid as a class, resulting in 21 kinds of amino acid compositions. We calculated the sequence of amino acid composition as follows:

$$\mathrm{AAC}\left(x_{a}\right)=\frac{\;number\;of\;amino\;acid\;x_{a}}{length\;of\;protein\;sequence}$$

where *x_a_* represents the 21 different amino acids.

#### 2.2.2. Shannon Entropy

In 1948, Claude E. Shannon proposed thermodynamic entropy in information theory to measure the expectations of a random variable for solving the quantization problem [32]. When a system is ordered, its entropy is low. By contrast, if a system is complex, its entropy is high. The Shannon entropy formula can be used to calculate the change rate in the data sets for each protein sequence in the amino acid residue position sequence [33]. p(x_i_) is the frequency of each amino acid sequence. The logarithm of p(x_i_) multiplied by p(x_i_) is determined to obtain the entropy as the feature coding.

$$H\left(X\right)=-\overset{I}{\underset{i=1}{\sum }}p\left(x_{i}\right)\log_{2}(p\left(x_{i}\right))$$

#### 2.2.3. Accessible Surface Area 

In protein folding, amino acid residues contain hydrophilic and hydrophobic charges. These residues are then folded into a 3D structure through their interactions. The hydrophobicity of residues is crucial to stabilize the protein structure. When proteins are in an aqueous solution, the hydrophobic amino acid side chains are embedded in the internal proteins to form a hydrophobic core and stable protein. The protein’s accessible surface area proposed by Lee and Richards [34] is used to study the hydrophobicity of protein molecules. The accessible surface area (ASA) indicates the contact area between the protein and solvent, which is divided into two states (i.e., exposure or embedded). The SAS web server [35] was used to obtain a sequence ASA to differentiate between monomer and multimer feature coding.

### 2.3. Model

In the study, we proposed a major component element known as the integration of classification to effectively use each feature and process the data classification problem. In most cases, the number of negative data (majority class) was higher than that of positive data (minority class), and the ratio of sizes between them usually exceeded three. Thus, for unbalanced data, we used R software in the bootstrap method for repeated sampling and then generated different subset data. According to the unbalance training data in the protein interaction problem processed by Deng et al. [36]. The majority class of information was subjected to random sampling, so that the majority class data number was equal or similar to the minority class data number in a certain subset. This step also ensured that the entire minority class data were retained in the overall dataset. Furthermore, Deng, et al. used voting strategy to integrate these submodels [36]. However, that strategy can’t be adopted when the submodels are an even number. Therefore, PClass selected the best learning model for each feature, which might come from different subset.

By integrating classification and bootstrap method, multimer negative information of training set was partitioned into the same or similar groups of positive data. Thus, each group with complex negative data were divided into *m* set, and each group positive data were integrated with the new classification of negative data to yield a new training data set.

**Figure 1:**
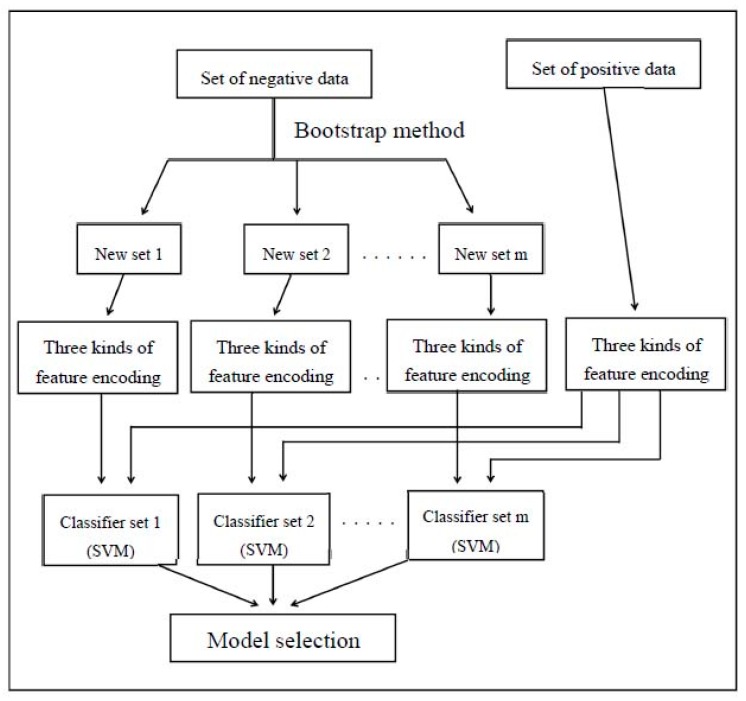
The flowchart of classifier evaluation. SVM: support vector machine.

The complex of each group was based on the classification of the new training data set for individual use of amino acid composition, entropy, and ASA for feature encoding. In addition, each group contains a *m* classification set and a support vector machine (SVM) classifier is a process for the classification. In order to assess the robustness of the SVM classifier, the tenfold cross validation method is used throughout the work. In Figure 1, the integration mechanism consists of two parts which the three best performing feature codings are performed to the feature modules of each group and the feature module is selected as a second layer to establish the integrating functions. Additionally, it was also used in conjunction with other machine learning methods to enhance the performance of each prediction system.

The monomers and dimers will not use the bootstrap method when the rates of positive are less than three. In this situation, the machine learning method will be used as an alternative. In contrast, trimers, tetramers, and other class subunits will be analyzed using the bootstrap method. That is, trimer data were divided into 20 negative and positive sets of data, and each group comprised three feature encodings. Tetramer data were divided into eight negative and positive sets of data integration, and each group included three feature encodings. Further, divided into 15 negative sets and a positive set of data integration in the other subunit types of information, there will be 15 of the group, each group having three feature encodings. To enhance the performance of each complex set classification system, the best MCC was selected as feature model through SVM confidence scores as the input of the second layer of integration mechanisms. Six kinds of machine learning methods (BayesNet, REPTree, LADTree, Kstar, MultilayerPerceptron, and RandomForest) were then used to choose the best machine learning with the best performance [37]. Furthermore, this study constructed a hierarchical testing by the best complex models from high to low individual performance when the unknown protein sequence was requested; a possible flowchart is shown in Supplementary Figure S1.

When the predict results for this multimer class and practical is also this multimer class, called as True Positive (TP). If the predicted results are for this multimer class but the actual result is not this multimer class, then the data are false positive (FP). If the predicted results are a nonpolymer class but actually a multimer class, then they are false negative (FN). Predictions for the nonmultimer class and actual nonmultimer class are called true negative (TN). Through the rules defined, the method accuracy and performance are assessed. MCC is used to test the positive and negative correlation, and its value is between [−1,1]. If the value of 1 represents an entirely correct forecast, then the weak value of −1 indicates that the forecast is opposite. The MCC can be calculated using the following formula:

$$MCC=\frac{\left(TP\times TN\right)-\left(FN\times FP\right)}{\sqrt{\left(TP+FN\right)\times \left(TN+FP\right)\times \left(TP+FP\right)\times \left(TN+FN\right)}}$$

Accuracy (ACC), which is used to assess the overall predictive ability of forecasting accuracy, is calculated as follows:

$$ACC=\frac{TP+TN}{TP+FP+TN+FN}$$

## 3. Results and Discussion

### 3.1. Training of Feature Encoding in First Layer

To understand the accuracy of the feature encoding prediction system, we used the SVM training data with tenfold cross-validation (Supplementary Figures S2–S6). Different features were used for the encoding of the results.

The trimer structure handle with imbalanced data on bootstrap method, through classifier evaluation can formation of twenty models, each model via SVM to select the best parameters. Among amino acid composition encoding, the model3 can reach a maximum MCC of 0.698. For the entropy encoding, the model3 can achieve, at its best, an MCC of 0.693. For ASA encoding, the model6 can reach an MCC of 0.363. In conclusion, we selected the best performance using the SVM confidence scores of amino acid composition and entropy as the feature models (Supplementary Figure S2).

The tetramer structure handle with imbalanced data on the bootstrap method, through classifier evaluation, can form eight models. Each model (via SVM) selected the best parameters among the amino acid composition encodings, with model1 reaching a maximum MCC of 0.742. For entropy encoding, model2 could attain the optimal MCC of 0.783. For ASA encoding, model5 reached the optimal MCC of 0.425. Ultimately, we chose the best model through SVM confidence scores of amino acid composition and entropy as the feature models (Supplementary Figure S3).

Other subunit classes were chosen to deal with unbalanced data on the bootstrap method. Through classifier evaluation, fifteen models were formed. Each model (via SVM) selected the best parameters among the amino acid composition encodings, with model15 reaching the best MCC of 0.757. For entropy encoding, model15 could reach the best MCC of 0.756. For ASA encoding, model14 could achieve the best MCC of 0.466. Finally, we selected the best model using SVM confidence scores of amino acid composition and entropy as the feature model (Supplementary Figure S4).

In the classification of trimers, tetramers, and other subunits, ASA feature coding did not achieve enhanced prediction accuracy. Thus, in the classification of monomers and dimers, we did not adopt ASA feature coding. For monomer data, feature encoding and machine learning methods with tenfold cross-validation were directly used to select the best machine learning method as a module. Machine learning methods include BayesNet, REPTree, LADTree, Kstar, MultilayerPerceptron, and RandomForest. Consequently, we selected the best machine learning method and performance, which were Kstar and MCC = 0.721, respectively. The best machine learning method for entropy encoding was Kstar, and the MCC was 0.724 (Supplementary Figure S5). For the dimers to the amino acid composition and entropy encoding, the best machine learning method was Kstar, and the MCC was 0.665 (Supplementary Figure S6).

### 3.2. Training of Integrate Method in Two Layer

To increase the classification efficiency and accuracy of the prediction system, the best performance model was selected in the first layer and integrated with the characteristics of other techniques (Supplementary Figures S7–S9). Given that ASA feature coding did not achieve enhanced prediction accuracy, we did not adopt ASA feature coding to establish the second layer.

We explored various machine learning methods to select the best method with the findings from the first layer. Then, the second layer used BayesNet, REPTree, LADTree, Kstar, MultilayerPerceptron, and RandomForest [38,39,40,41,42]. The trimeric class used machine learning methods, and the best machine learning method was selected (Supplementary Figure S7). The trimer data used MultilayerPerceptron to achieve the best performance of MCC = 0.808, whereas other machine learning techniques reached an MCC above 0.7. Compared with the first layer of the amino acid composition and entropy, MCC was 0.654 and 0.610 (Supplementary Figure S10). Thus, the performance increased from 13% to 19%.

For the tetramer class, we used machine learning methods to pick the best learning algorithms. Show in Supplementary Figure S8. It can be seen that for the tetramer data we used Kstar to achieve the best performance of MCC 0.764. Other machine learning performance can reach an MCC above 0.72. Compared with the first layer of the amino acid composition and entropy, MCC was 0.63 and 0.61 (Supplementary Figure S11); thus, the performance increased from 11% to 15%. Other subunits were subjected to different machine learning methods, whereas other subunit information used REPTree to achieve the best performance of MCC = 0.773 (Supplementary Figure S9).

Other machine learning techniques reached an MCC above 0.73. Compared with the first layer of the amino acid composition and entropy, MCC was 0.52 and 0.57 (Supplementary Figure S12); thus, the performance increased from 22% to 25%. Therefore, we utilized different machine learning methods and chose the best one, integrating feature as a combination. For trimer, tetramer, and the other subunit class can enhance the prediction of the system performance and accuracy.

**Figure 2:**
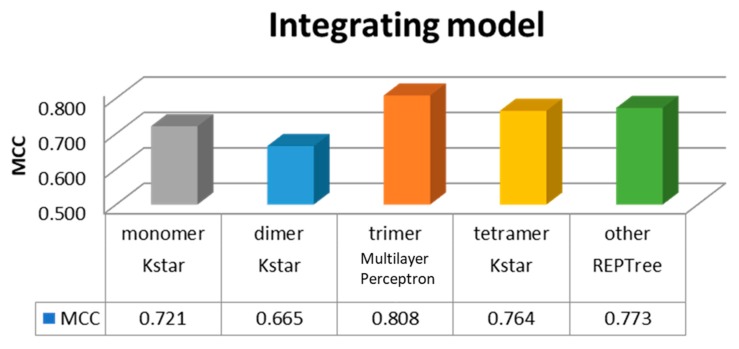
Different complexes and their best performance integrated module. MCC: Matthews correlation coefficient.

Divided into five categories in the study, monomer, dimer, trimer, tetramer, and other subunits of class, each class establish a module, and selection of the best performance through machine learning. Finally, we selected the overall module, as shown in Figure 2. Hence, the model order was the trimer with MCC = 0.808, followed by the other subunits with MCC = 0.773, tetramer with MCC = 0.764, monomer with MCC = 0.721, and dimer with MCC = 0.665.

### 3.3. Bootstrap Method Compare with Other Method

The trimer, tetramer, and other class subunits must be data processed. Due to negative information and positive information ratio greater than three, the bootstrap method was used to deal with unbalanced data to achieve the best performance classification system. In this study, we randomly selected negative data 10 times for making positive data and negative data quantity of the same number, called the random method, and to verify that the bootstrap method can make the prediction system for optimal performance.

As shown in Table 3, the average performance was 0.696 using trimer data by the bootstrap method for imbalanced data and first-layer feature models. Trimer data using random method exhibited an average performance of only 0.676. Thus, the trimer structure data obtained by the bootstrap method could improve the prediction ability of the system. Tetramer data via the bootstrap method for imbalanced data through first-layer feature models demonstrated an average performance of 0.741. Tetramer data via the random method yielded an average performance of 0.727. For this reason, tetramer structure data from the bootstrap method could improve the prediction ability of the system. Other subunits for imbalanced data by the bootstrap method through first-layer feature models revealed an average performance of 0.757, whereas those using the random method indicated an average performance of 0.738. As a result, the other subunits via the bootstrap method improved the prediction ability of the system.

### 3.4. Case Study

Using the transcription factor sequence data, we selected nine transcription factor sequences to predict the dimer structure. Using the viral infection-associated glycoprotein data, we selected nine virus-infection-associated glycoprotein sequence data to predict the trimer structure. All protein database (PDB) IDs of selected proteins were shown in supplementary dataset. In this study, we accurately predicted three virus-infection-associated glycoproteins belonging with a trimer sequence. The three trimer sequences belonged to human immunodeficiency virus (HIV) type I-associated glycoprotein gp41. The remaining six trimer sequence data belonged to the trimeric HIV information, but they may be related to glycoprotein gp120 virus or other membrane fusion proteins. Thus, the classification system of trimers could accurately predict the virus-infection-associated glycoprotein gp41 sequence. In the prediction of the dimeric transcription factor sequence, the proposed system could accurately predict the sequences belonging to the dimeric transcription factor.

## 4. Conclusions

This study aimed to conduct feature encoding and integration mechanisms for classifying quaternary structures. For this purpose, we designed the architecture of a two-layer machine learning technique. Two objective layers namely the bootstrap method to classify unbalanced data and selected the optimum parameters of the SVM feature module are introduced to be used along with other machine learning methods to enhance the prediction performance. The first layer used a variety of feature encoding via SVM machine learning to find the best parameters for each set as a model. In addition, each model has a m-predicted performance for selecting the best forecasting performance. The trimer, tetramer, and other subunits were selected as feature encoding of amino acid composition and entropy for an overall prediction performance above 0.7 and ASA of 0.3. Thus, in the first layer of feature coding, we selected the amino acid composition and entropy to integrate the prediction performance as the feature module. In particular, the second layer used machine learning methods, and the selection of the optimum parameters of the SVM feature module in the first layer. Effectiveness of proposed machine learning methods is shown by comparing it with first layer. That is, the second layer of construction combines the first-layer module to integrate mechanisms and the best machine learning method was selected to improve the prediction performance. Indeed, this system can be improved over 10% in MCC.

In this work, we analyzed the performance of our classification system using transcription factors with a dimer structure and virus-infection-associated glycoprotein with a trimer structure. There was a superiority of two-layer machine learning to predict and classify protein quaternary structures in dimers, trimers, tetramers, and other subunits. In addition to predicting the protein quaternary structure on the polymer structure, the interactions between the coiled-coil position and structure, homologous polymer and heterologous polymer structure, and parallel and antiparallel polymer structures may be investigated to establish a human polymer molecular database.

Finally, we provided an advanced web tool to users for the complete single-chain sequence of the protein quaternary structure. Results showed a sequence of quaternary structure belonging to the protein monomer, protein dimer, trimer proteins, tetramer protein, or other subunits.

## Figures and Tables

**Table 1:** Training set and independent test set basis on the year to do classification.

Training Set		Independent Test Set
**Monomer**	11,638	1513
**Dimer**	8570	1005
**Trimer**	1231	119
**Tetramer**	2764	282
**Other**	1527	176

**Table 2:** Positive and negative data of training set and independent test.

Training Set			Independent Test Set	
	Positive	Negative	Positive	Negative
**Monomer**	11,638	14,092	1535	1582
**Dimer**	8570	17,160	1005	2112
**Trimer**	1231	24,499	119	2998
**Tetramer**	2764	22,966	282	2835
**Other**	1527	24,203	176	2941

**Table 3:** Bootstrap method compared with random method.

	Trimer	Tetramer	Other
Bootstrap	0.696	0.741	0.757
Random	0.676	0.727	0.738

## Supplementary Materials

The following are available online at http://www.mdpi.com/2073-4425/9/2/91/s1, File F1: PClass_SD.zip, Supplementary Figures S1–S12; dataset: ds.txt.
