# Supplementary material for: PClass: Protein Quaternary Structure Classification by Using Bootstrapping Strategy as Model Selection
Source: Genes (Basel). 2018 Feb 14;9(2):91. doi: 10.3390/genes9020091 (PMC5852587; doi:10.3390/genes9020091)
Supplement: Supplementary file 1 [file genes-09-00091-s001.zip › PClass Supplementary data_ywchu_01.docx]

# Supplementary data


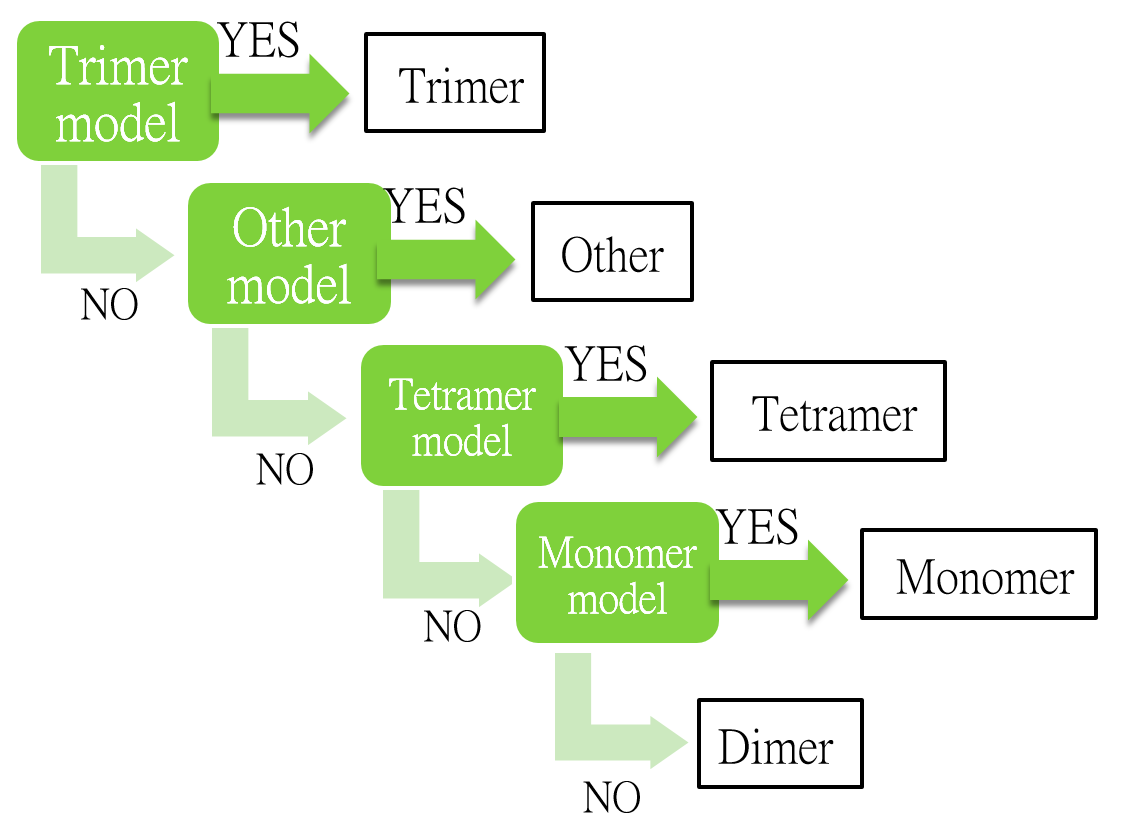


Supplementary figure 1: An example flowchart of the hierarchical testing process for an unknown protein sequence

Supplementary figure 2: Prediction performance of amino acid composition and entropy are above 70% in trimer.

Supplementary figure 3: Prediction performance of amino acid composition and entropy are above 70% in tetramer.

Supplementary figure 4: Prediction performance of amino acid composition and entropy are above 70% in other subunits class.

Supplementary figure 5: The prediction accuracy of dimer by KStar and Random Forest can reach above 60%.

Supplementary figure 6: The prediction accuracy of monomer by KStar and Random Forest can reach above 70%.

Supplementary figure 7: To compare different machine learning algorithms by amino acid composition and entropy in the second layer of PClass in trimer.

Supplementary figure 8: To compare different machine learning algorithms by amino acid composition and entropy in the second layer of PClass in tetramer.

Supplementary figure 9: To compare different machine learning algorithms by amino acid composition and entropy in the second layer of PClass in other subunits class.

Supplementary figure 10: To compare different machine learning algorithms in the first layer of PClass in trimer.

Supplementary figure 11: To compare different machine learning algorithms in the first layer of PClass in tetramer.

Supplementary figure 12: Other first layer compare with machine learning methods.
